# Investigation on the Underlying Mechanisms of the Mechanical and Electrical Enhancement of Nano-SiO_2_-Doped Epoxy Resins: A Molecular Simulation Study

**DOI:** 10.3390/molecules30142960

**Published:** 2025-07-14

**Authors:** Kunqi Cui, Yang Wang, Wenchao Yan, Teng Cao, Yan Du, Kai Wu, Li Guo

**Affiliations:** 1 School of Electronics and Information, Xi’an Polytechnic University, Xi’an 710048, China; cuikunqi_xpu@163.com (K.C.); ywchao@stu.xpu.edu.cn (W.Y.); tengcao@stu.xpu.edu.cn (T.C.); duyan@xpu.edu.cn (Y.D.); 2 Xi’an Key Laboratory of Interconnected Sensing and Intelligent Diagnosis for Electrical Equipment, Xi’an Polytechnic University, Xi’an 710048, China; 3 State Key Laboratory of Electrical Insulation and Power Equipment, Xi’an Jiaotong University, Xi’an 710049, China; wukai@xjtu.edu.cn; 4 Central Research Institute, TBEA Science & Technology Investment Co., Ltd., Tianjin 301700, China

**Keywords:** epoxy resins, SiO_2_ nanocomposites, interface, Molecular Simulation, mechanical properties, electronic properties

## Abstract

As a key insulating material in power equipment, epoxy resins (EP) are often limited in practical applications due to space charge accumulation and mechanical degradation. This study systematically investigates the effects of SiO_2_ nanoparticle doping on the electrical and mechanical properties of SiO_2_/EP composites through molecular dynamics simulations and first-principles calculations. The results demonstrate that SiO_2_ doping enhances the mechanical properties of EP, with notable improvements in Young’s modulus, bulk modulus, and shear modulus, while maintaining excellent thermal stability across different temperatures. Further investigations reveal that SiO_2_ doping effectively modulates the interfacial charge behavior between EP and metals (Cu/Fe) by introducing shallow defect states and reconstructing interfacial dipoles. Density of states analysis indicates the formation of localized defect states at the interface in doped systems, which dominate the defect-assisted hopping mechanism for charge transport and suppress space charge accumulation. Potential distribution calculations show that doping reduces the average potential of EP (1 eV for Cu layer and 1.09 eV for Fe layer) while simultaneously influencing the potential distribution near the polymer–metal interface, thereby optimizing the interfacial charge injection barrier. Specifically, the hole barrier at the maximum valence band (VBM) after doping significantly increased, rising from the initial values of 0.448 eV (Cu interface) and 0.349 eV (Fe interface) to 104.02% and 209.46%, respectively. These findings provide a theoretical foundation for designing high-performance epoxy-based composites with both enhanced mechanical properties and controllable interfacial charge behavior.

## 1. Introduction

Epoxy resin (EP), as a core material for insulation systems in electrical power equipment, plays a pivotal role in ensuring long-term operational reliability [1,2,3]. With the continuous increase in voltage levels of power systems, insulation materials are required to withstand more stringent electric field environments, presenting new challenges for the dielectric properties of EP composites [4,5]. Under high electric fields, the migration of electrons can lead to molecular chain damage and the accumulation of space charge, resulting in a significant deterioration of insulation performance [6,7]. Among various modification methods, nanoparticle doping has proven to be particularly effective. Recent studies have shown that interfacial traps introduced by doping at the polymer–metal interface can effectively regulate charge transport behavior across this interface. Research by Yu et al. confirmed that introducing ZnS:O nanoparticles, in which a part of S in ZnS was substituted by O, with specific electronic structures into polymer matrix can effectively confine space charges and improve its insulation properties [8]. Yang’s team found that even without surface modification of diamond nanofillers, optimized interfacial structures between the doping diamond and the EP matrix can suppress charge accumulation and enhance e dielectric strength [9]. These findings highlight the critical role of nanoparticle doping in improving insulation performance of EP. Further studies reveal that the energy level structure and barrier characteristics at polymer–metal interfaces are key factors influencing electron migration behavior across this interface [10,11,12]. Ren et al. theoretically demonstrated that the formation of electric double layers (EDLs) at different polymer–metal (Cu or Al) interfaces significantly affects interface potential barriers [13]. In summary, the modulation of interfacial electronic properties through nanoparticles represents an effective approach for enhancing the electrical characteristics of insulating materials, although the underlying mechanisms require further investigation.

The thermo-mechanical properties of insulation materials are equally crucial in power equipment applications. During long-term operation, materials must endure multiple environmental stresses, including temperature variations, mechanical vibration loads, and thermal aging [14]. It may cause adverse consequences such as EP molecular chain breakage and cross-linking degradation, cyclic load fatigue, and glass transition temperature (Tg) drift. Similarly, doping nanoparticles in EP is also an effective means to optimize the above performance: Wang et al. found that the proper concentration of Ag nanoparticle doping can effectively enhance the thermo-mechanical performance of EP-based composites [15]. Luo’s team achieved synergistic enhancement of thermal conductivity and mechanical properties of EP by doping silver nanoparticles into functionalized boron nitride nanoplates (AgBNs) [16]. Sallal et al. systematically investigated the enhanced mechanical properties of co-doping Al_2_O_3_ and CaO nanoparticles into EP polymer blends [17]. Additionally, other researchers concluded that the thermos-mechanical performance improvement of polymer nanocomposites primarily stems from three key factors: enhanced interfacial chemical interactions, optimized filler distribution, and improved matrix-nanofiller thermo-mechanical compatibility [18,19,20]. The development of this EP nanocomposite material provides an important technical approach for achieving next-generation insulating materials with pronounced mechanical properties that can adapt to complex working conditions.

Although the dielectric and electrical properties of EP nanocomposites have been extensively studied [21,22,23], the influence mechanisms of nanoparticles on its local morphology and electrical performance, particularly at the atomic scale, remain insufficiently understood. To address these fundamental scientific issues, this study focuses on investigating the mechanical properties of nano-SiO_2_ doped EP as well as the charge transfer behavior and potential mechanisms at different Metal–EP interfaces. Therefore, we established atomic models of nanoscale EP/SiO_2_ composite systems and their Metal–EP interfaces with or without nano-SiO_2_ doping, employing a combined approach of molecular dynamics (MD) simulations and density functional theory (DFT) to systematically study the underlying mechanisms of the enhanced mechanical and electrical properties of EP by nano-SiO_2_ doping. This dual-scale approach allows us to comprehensively investigate both the mechanical and electronic properties of EP/SiO_2_ nanocomposites and metal-EP interfaces. MD simulations effectively capture the mesoscale behavior, such as polymer chain dynamics and bulk mechanical properties, while DFT provides atomic-level insights into electronic structure, charge transfer mechanisms, and defect states at interfaces. By integrating these methods, we bridge the gap between macroscopic material performance and microscopic interactions, offering a more complete understanding of how SiO_2_ doping modifies EP’s properties. This work provides theoretical insights for the design of high-performance epoxy-based composites with both enhanced mechanical properties and controllable interfacial charge behavior.

## 2. Results and Discussion

### 2.1. Molecular Modeling and Simulation Details

**Figure 1:**
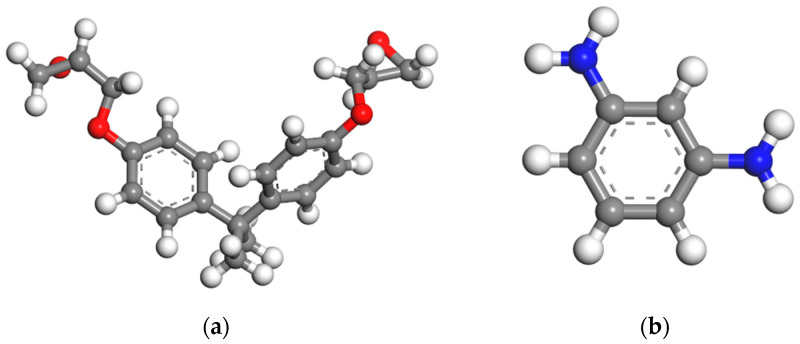
Molecular structure of key components for EP crosslinking: (**a**) DGEBA (polymerization = 1), (**b**) MPD. The gray, white, blue, and red spheres represent the C, H, N, and O atoms, respectively.

This study investigates the mechanical properties of the EP/SiO_2_ nanocomposite and the interfacial electronic behavior of the Metal–EP interface with or without nano-SiO_2_ doping. Therefore, two sets of atomic models, EP/SiO_2_ nanocomposite models and Metal–EP interfacial models (Cu or Fe for metal layer), were constructed using molecular dynamics approach in the Materials Studio (2020) software from BIOVIA (San Diego, CA, USA). The CIF (Crystal Graphic Information Files) of all models have been placed in the Supplementary Materials. According to previous studies, EP was modeled mainly by crosslinking Bisphenol A diglycidyl ether (DGEBA) as the matrix (polymerization = 1) with phenylenediamine (1,3-phenylenediamine, MPD) as the curing agent [24,25]. Figure 1 shows the molecular structure characteristics of the key components in the system.

#### 2.1.1. EP/SiO_2_ Nanocomposite

The EP/SiO_2_ nanocomposite atomic model in this study comprise 20 DGEBA polymer chains (degree of polymerization = 1), 10 MPD molecules, and a hydroxyl-functionalized SiO_2_ nanosphere (diameter = 4 Å). For the doped system, the weight ratio of SiO_2_ is approximately 6.9%. Owing to computational limitations, we used SiO_2_ nanoparticles smaller than the standard sizes found in the literature, as nanoparticle size has relatively little effect on the polymer matrix’s electrical properties compared to doping concentration [26].

The three-dimensional amorphous cell model was constructed by using the COMPASS II force field in the Amorphous Cell module of Material Studio (2020) followed by structural optimization via the Forcite module. In this paper, the calculation results obtained using the COMPASS II force field is largely consistent with those derived from the COMPASS III force field. Consequently, the COMPASS II force field is employed in this study [27,28]. Energy minimization was performed sequentially using the steepest descent algorithm and conjugate gradient method, both with a maximum iteration limit of 3000. System equilibration involved sequential NVT (density = 1.0 g/cm^3^) and NPT (1 atm) ensemble simulations (duration = 100 ps, timestep = 1 fs, temperature = 300 K), employing the COMPASS II force field with Andersen thermostat and Berendsen barostat controls.

**Figure 2:**
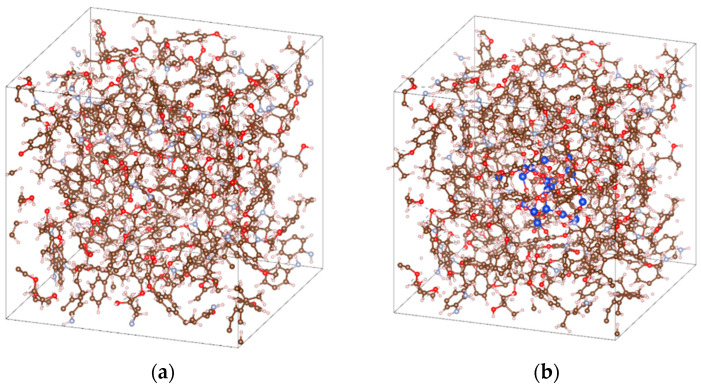
EP crosslinking models: (**a**) EP model, (**b**) EP/SiO_2_ nanocomposite model. The white, brown, sky-blue, red, and blue spheres represent the H, C, N, O and Si atoms, respectively.

The crosslinking simulation capitalized on the chemical reactivity between epoxy and amine groups: the partially positive terminal carbon atoms of epoxy chains react with electronegative amine nitrogens, inducing nitrogen attack on carbon atoms on DGEBA chains with concomitant hydrogen abstraction, followed by hydroxyl formation via hydrogen–oxygen bonding. Each amine nitrogen can simultaneously react with two carbon atoms on DGEBA chains, enabling three-molecule crosslinking (as shown in Figure 2). Given the higher reactivity of terminal carbons, the DGEBA: MPD molar ratio was fixed at 2:1. Crosslinking simulations were conducted at 300 K and atmospheric pressure using the NPT ensemble and COMPASS II force field, with Perl scripts controlling the reaction process to achieve 90% crosslinking (approaching experimentally values). Post-crosslinking, the system underwent 100 ps NPT equilibration at 500 K until density fluctuations stabilized, followed by stepwise cooling to 300 K in 50 K increments (100 ps per temperature under NPT conditions), concluding with 500 ps NPT equilibration at 300 K. The final equilibrated model (as shown in Figure 2) successfully represents an epoxy composite system with well-defined network structure, providing a reliable atomic-scale foundation for subsequent property investigations.

#### 2.1.2. Interfacial Models Between EP/SiO_2_ Nanocomposite Layer and Metal Layer (Cu or Fe)

The Metal/EP (with or without nano-SiO_2_ doping) atomic models were constructed as follows: Initially, the cubic unit cells of Cu and Fe were structurally optimized using the Forcite module in the Materials Studio (2020) software, followed by the construction of 4 × 4 × 4 supercells along the (1 0 0) plane with lattice parameters of 11.36 Å × 11.36 Å and 11.45 Å × 11.45 Å, respectively. Secondly, we built an EP layer for the interface model. It contains 10 DGEBA polymer chains (degree of polymerization = 1) and 5 MPD molecules. To ensure lattice matching between the metal and EP layers, the cross-sectional lattice dimensions of the EP structure were adjusted accordingly. By integrating the optimized metal layers with the EP layers, two distinct interfacial structures were successfully established: Cu (1 0 0)/EP and Fe (1 0 0)/EP. To investigate the doping effects, SiO_2_ nanospheres were incorporated into the EP layer to form Metal/EP@SiO_2_ composite interfacial systems.

**Figure 3:**
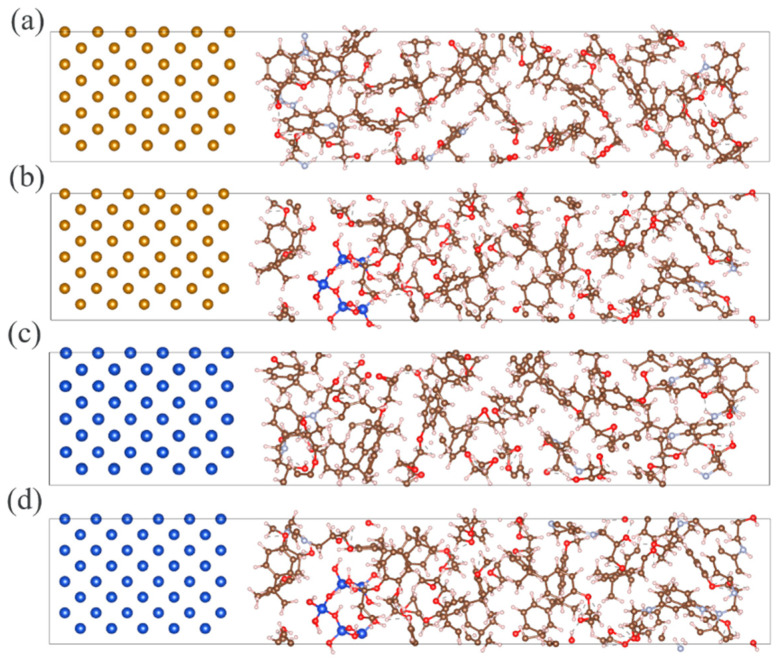
Different Metal/EP interfacial atomic structures: (**a**) Cu/EP; (**b**) Cu/EP@SiO_2_; (**c**) Fe/EP; (**d**) Fe/EP@SiO_2_.

All calculations were performed using the VASP 6.1.0 software (Vienna Ab initio Simulation Package, Vienna, Austria), with the PBE-GGA functional [29,30,31,32]. The plane-wave cutoff energy was set to 400 eV, and the self-consistent field convergence criterion was established at 10^−4^ eV. A 10 Å vacuum layer was introduced above the metal (1 0 0) surfaces, and a 2 × 2 × 1 k-point mesh was employed for Brillouin zone sampling. The final optimized atomic configurations, as illustrated in Figure 3, provide a reliable atomic modeling foundation for subsequent investigations of interfacial electronic properties between Metal/EP (with or without nano-SiO_2_ doping) interfaces.

### 2.2. Mechanical Properties of EP/SiO_2_ Nanocomposite

To further investigate the contribution of molecular thermal motion of EP atoms near SiO_2_ nanoparticles to local density fluctuations, we calculated the diffusion characteristics of EP atoms in different regions based on the average of the last 100 ps of NPT ensemble molecular dynamics trajectories, as described above. The diffusion behavior and thermal motion of small molecules or atoms can be characterized by the mean square displacement (*MSD*) [33,34], which represents the average squared distance deviation of target particles from their initial positions at time 0 to their current positions at time *t*. By measuring the *MSD* evolution along particle trajectories during thermal dynamic processes, we can determine whether the particles undergo directional or non-directional diffusion due to molecular thermal motion, expressed as follows [35]:

(1) $$MSD=\langle {\left|{\vec{r}}_{i}\left(t\right)-{\vec{r}}_{i}\left(0\right)\right|}^{2}\rangle$$

where ${\vec{r}}_{i\;}(t)$ and ${\vec{r}}_{i}\;(0)$ represent the position vectors of the *i*-th atom at time *t* and initial time 0 in each timestep, respectively. The diffusion coefficient (*D*) [30] can be obtained by fitting the *MSD* curve according to Equation (2) during MD simulations.

(2) $$D=\frac{1}{6N}\underset{t\to \infty }{\lim}\frac{d}{dt}\sum_{i=1}^{n}{\left|{\vec{r}}_{i}\left(t\right)-{\vec{r}}_{i}\left(0\right)\right|}^{2}=\frac{1}{6}a$$

**Figure 4:**
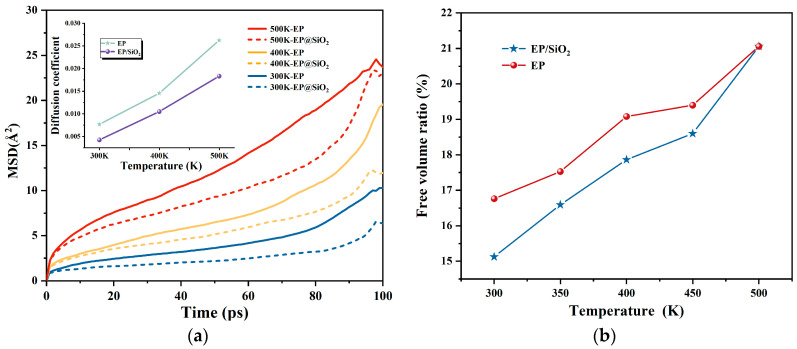
*MSD*, Diffusion coefficient and free volume ratios calculations for EP/SiO_2_ nanocomposite models at different temperatures: (**a**) mean square displacement and Diffusion coefficient, and (**b**) Free volume ratio.

Here, *N* denotes the number of target molecules in the model, *n* represents the number of timesteps, and *a* is the slope obtained from linear fitting of the *MSD* curve. The free volume ratio refers to the ratio of the volume not occupied by molecules in a material to the total occupied volume. It affects the movement of molecules and the glass transition of materials. When the free volume decreases to a certain extent, the movement of molecular chains is restricted, thereby causing changes in the properties of the material. The *MSD* of EP atoms at different temperatures was calculated and plotted in Figure 4a.

Figure 4a presents the calculation results of the mean squared displacement (*MSD*). Specifically, the colored solid lines in the main plot denote the *MSD* calculation results, whereas the stars marked in various colors in the subplot indicate the diffusion coefficients corresponding to different systems or temperatures. Comparative analysis reveals that, at identical temperatures, the *MSD* values of the undoped system are significantly higher than those of the doped system. This observation mainly stems from mechanisms such as the physical interaction between the functional groups grafted on the surface of nanoparticles and the polymer chains, which restricts the movement of molecular chains and strengthens the cross-linked network [36,37,38], unequivocally confirming that the incorporation of SiO_2_ nanoparticles effectively suppresses the thermal motion of epoxy resin molecular chains. As temperature increases, the diffusion coefficient of EP molecules gradually rises. However, across all calculated temperatures, the diffusion coefficient of EP molecules in the SiO_2_ nanoparticle-doped system remains lower than that in the pure EP system.

Concurrently, as the temperature rises, both the *MSD* values and free volume fractions for all systems show an increasing trend, reflecting the enhanced molecular chain mobility promoted by elevated temperatures. Notably, the free volume analysis results, as shown in Figure 4b, corroborate the diffusion coefficient findings, verifying a direct relationship between molecular chain mobility and the internal free volume ratio of the material. This phenomenon could be attributed to the increase in the local density of molecular chains in the interface region of the doped system, which leads to a decrease in the free volume of the system [39,40].

**Figure 5:**
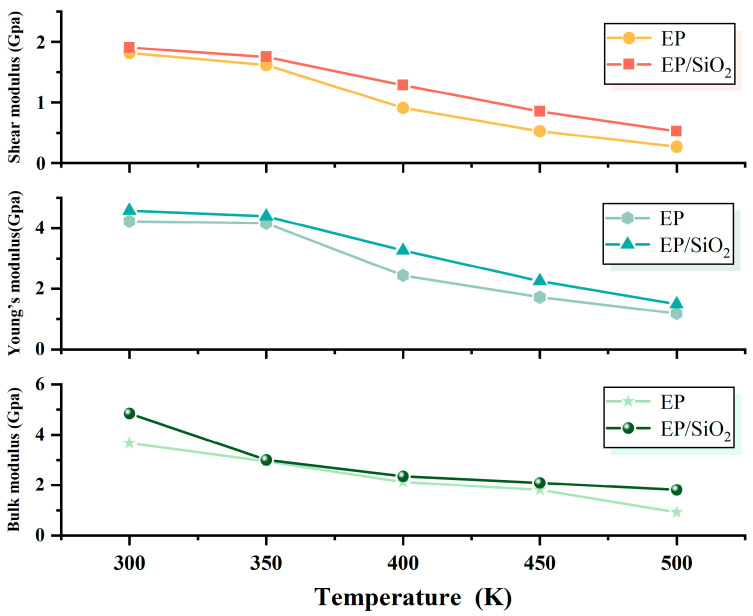
Mechanical properties of EP/SiO_2_ nanocomposite and pure EP as a function of temperature: shear modulus (**Top** panel), Young’s modulus (**Middle** panel), and bulk modulus (**Bottom** panel).

As shown in Figure 5, the results of Young’s modulus, bulk modulus, and shear modulus obtained through molecular dynamics simulations indicate that the incorporation of SiO_2_ nanoparticles significantly enhances the mechanical properties of EP-based composites [41]. Compared to pure EP systems, the doped systems exhibit superior mechanical performance under all tested temperature conditions. The increase in elastic modulus suggests an enhanced ability of the material to resist deformation. Furthermore, the improvement in bulk modulus reflects an optimization in the material’s resistance to volumetric compression. Lastly, the rise in shear modulus confirms a heightened capacity of the material to withstand shear deformation. Notably, this enhancement is maintained across various temperature conditions, indicating that SiO_2_ doping not only improves the mechanical properties at room temperature but also imparts good thermal stability to the composite materials. This enhancement effect may be attributed to several mechanisms: (1) the interfacial interactions between nanoparticles and EP matrix restrict molecular chain mobility; (2) the rigid characteristics of nanoparticles help distribute external stress more effectively.

After confirming that the incorporation of SiO_2_ nanoparticles significantly enhances the mechanical properties of EP-based composites, we further investigated the electronic properties at the interface between this composite system and the metals. Firstly, the practical application of polymer composites often involves contact with metal electrodes; moreover, interfacial charge behavior directly influences the electrical insulation performance and long-term reliability of high-voltage power equipment. Secondly, the introduction of nanoparticles may modulate charge injection and transport processes by altering the electronic structure at the interface. To this end, this study systematically examines the interfacial electronic properties between the composite system and typical metals layers (Cu and Fe), focusing on analyzing changes in energy level alignment, potential distribution, and interfacial potential barrier before and after nano-SiO_2_ doping.

### 2.3. Electronic Properties Across Different Metal/EP Interfaces with/Without Nano-SiO_2_ Doping

**Figure 6:**
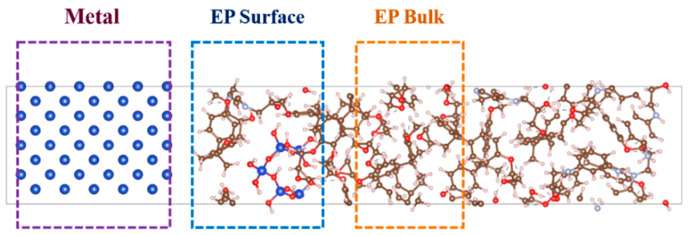
Three different regions in Metal/EP interfacial structures, namely Metal, EP Surface, and EP Bulk, respectively.

To elucidate the electronic characteristics at Metal/EP interfaces, this study systematically investigated the electronic density of states (DoS) of both nano-SiO_2_-doped and undoped EP interfaces with Cu and Fe using first-principles calculations. To systematically evaluate the distinct contributions of the metal, nanocomposite, and epoxy (EP) bulk to defect states near the interface, we computed the local density of states (LDOS) across three key interfacial regions: (1) the metallic zone, (2) the interfacial “EP surface” region containing the nanocomposite, and (3) the sub-surface “EP bulk” region, as illustrated in Figure 6.

**Figure 7:**
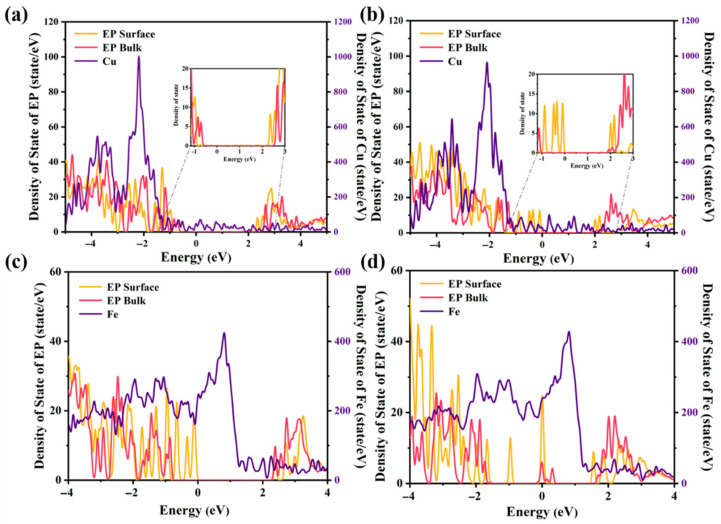
Local and Projected DoS of four Metal/EP interfacial models with or without SiO_2_ nanoparticles: (**a**) Cu/EP; (**b**) Cu/EP@SiO_2_; (**c**) Fe/EP; (**d**) Fe/EP@SiO_2_. *E*_Fermi_ = 0 eV for all the interfacial structures. The purple numbers on the right represent the vertical coordinate scale of the metal layer (Cu/Fe).

Figure 7 clearly reveals that in SiO_2_-doped systems, the LDoS of the interfacial “EP surface” region exhibits pronounced defect states within the bandgap, which are completely absent in un-doped systems. This phenomenon primarily results from SiO_2_ nanoparticle doping, which reconstructs the interfacial electronic structure through the introduction of deep defect states. Additionally, the hydrogen-bond network formed between surface hydroxyl groups (-OH) of SiO_2_ nanoparticles and epoxy molecular chains may serve as a complementary mechanism for defect state formation. It is notable that multiple defect states have emerged near the mid-gap of the doped interface structure, which can serve as “springboards” to increase the carrier mobility in the polymer, thereby reducing the accumulation of space charges at this interface [42,43].

Regarding charge transfer mechanisms, theoretical predictions suggest two dominant pathways: (1) direct tunneling, where electrons traverse the barrier from the insulator valence band to the metal conduction band [44], and (2) defect-assisted hopping via gap states. This study finds that the Fermi levels of all interface systems reside stably within the bandgap, unequivocally confirming that interfacial charge transfer primarily occurs through defect-assisted hopping [45,46]. Furthermore, comparative analysis shows that the DoS values near the Fermi level for both metals exceed those of epoxy resin by orders of magnitude, directly reflecting the superior electron exchange capability of metal electrodes that dominates interfacial charge transfer processes.

To systematically investigate the influence of SiO2 doping on interfacial potential distribution, this study calculated the electrostatic potential profiles of four representative interface structures based on first-principles calculations. The Cu-1s and Fe-1s core level was selected as the reference energy level for potential alignment for different models due to its stable electron binding energy that remains unaffected by interfacial charge transfer processes [47].

**Figure 8:**
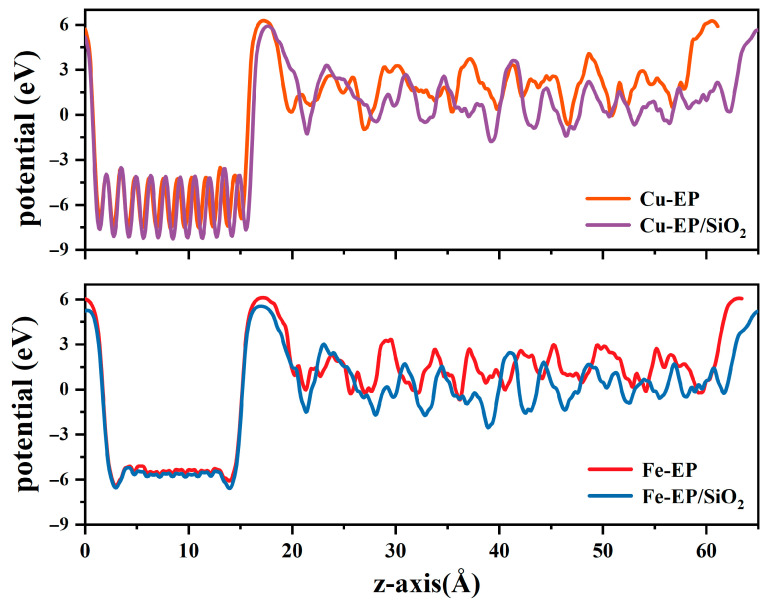
Potential distribution along the z-axis of four Metal/EP interfacial structures.

The calculated potential distributions are presented in Figure 8, showing potential variation ranges of [0, 15.59] Å for Cu layers and [0, 13.88] Å for Fe layers. Notably, the EP layers behind both metal interfaces exhibit significant potential oscillations. To quantify this characteristic, we specifically calculated the average potential values in the bulk region along the Z-axis direction with stable oscillations (within the range of [18–60] Å), as summarized in Table 1 and Table 2. These results clearly demonstrate that SiO_2_ doping effectively reduces the average potential of the EP matrix while significantly altering the potential distribution near the Metal/EP interfaces.

**Figure 9:**
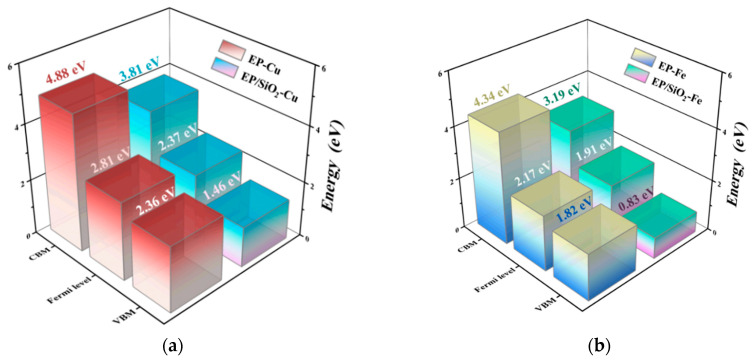
Interfacial potential barriers between Metal and EP in different interfacial structures. (**a**) Cu/EP with or without SiO_2_, (**b**) Fe/EP with or without SiO_2_.

To further elucidate the interfacial electronic characteristics between SiO_2_-doped EP and metals, we determined the interface potential barriers according to the following formulation:

(3) $$E_{sVBM}^{i}=E_{VBM}^{i}-E_{Cu/Fe-1s}^{i}$$

(4) $$E_{sCBM}^{i}=E_{CBM}^{i}-E_{Cu/Fe-1s}^{i}$$

(5) $$\Delta E_{sVBM}^{i}=E_{sVBM}^{i}-E_{sVBM}^{1}$$

(6) $$\Delta E_{sCBM}^{i}=E_{sCBM}^{i}-E_{sCBM}^{1}$$

where VBM and CBM denote the valence band maximum and conduction band minimum of bulk EP, respectively, while Cu/Fe-1s represents the 1s core-level energies of the respective metals. The core-level-corrected VBM and CBM are designated as sVBM and sCBM. Four representative interface configurations (*i* = 1–4) were investigated: Cu/EP, Cu/EP@SiO_2_, Fe/EP, and Fe/EP@SiO_2_. To address the bandgap underestimation issue of PBE functional, we employed the PBE0 hybrid functional (with a 2 × 2 × 2 k-point mesh and 400 eV cutoff energy) for calculating average potentials and band edge positions [48]. Considering the computational limitations for large-scale interface models, we adopted the band alignment method to determine the Metal/EP interface barriers. Structural optimization was performed using the following lattice parameters: Cu (2.84 Å cubic unit cell), Fe (2.86 Å cubic unit cell), and EP systems (15 Å cubic supercell). Based on the PBE0-calculated Fermi levels of metals and band positions of EP, combined with the reference potential values from Table 1 and Table 2, we ultimately obtained the interface barrier distribution results shown in Figure 9, which provides important insights into the charge transport mechanisms.

The analysis of interface barrier indicates that the incorporation of SiO_2_ significantly alters the electronic structure characteristics at the Metal/EP interface after an average potential correction. For the Cu/EP@SiO_2_ and Fe/EP@SiO_2_ systems, the reduction in metal interface potential energy aligns with previous findings on potential distribution. Notably, in both metallic interface structures, there is a pronounced decrease in electron barriers at the conduction band minimum (CBM) following doping; Specifically, for Cu/EP@SiO_2_ and Fe/EP@SiO_2_ systems, the metal interface potential barriers decreased by approximately 30.43% and 41.74%, respectively. These changes may promote electron injection and exacerbate the risk of space charge accumulation and local electric field distortion, thereby leading to dielectric failure [49,50,51,52]. However, there is a significant increase in hole barriers at the valence band maximum (VBM) post-doping, rising from initial values of 0.448 eV (Cu interface) and 0.349 eV (Fe interface) to increases of 104.02% and 209.46%, respectively, which is much larger than the decrease in electron barriers at the CBM. Overall, in these Metal/EP interfacial systems, the enhancement of electronic properties due to the increased hole barriers near VBM after doping is more significant than the detrimental effects caused by electron barriers at CBM. This study provides important theoretical guidance for designing high-performance nanocomposite insulating materials through quantitative calculations of SiO_2_-doped interfacial barriers. By enhancing electronic coupling between metals and epoxy resins, this interfacial engineering strategy holds promise for significantly improving the overall insulation performance of composite materials.

## 3. Conclusions

This study systematically investigates the regulation mechanism of SiO_2_ nanoparticle doping on EP composite properties through various computational simulations, combining molecular dynamics and first-principles calculations. The results demonstrate that SiO_2_ doping simultaneously enhances both mechanical properties and interfacial electrical properties. Mechanically, the nanoparticles significantly improve material strength by reinforcing interfacial interactions between the nanoparticle and the EP matrix, leading to the enhancement of its shear modulus, Young’s modulus, and bulk modulus. Electrically, nano-SiO_2_ doping improves the electrical performance of EP via dual mechanisms: (a) raise the hole injection barrier to suppress charge accumulation. At the interface between EP and Cu, SiO_2_ doping increased the barrier by approximately 0.54 eV. For Fe, the hole injection barrier increased by 0.73 eV, and (b) introduced multiple defect states in EP that promote charge dissipation while mitigating space charge accumulation. This unique synergistic compensation mechanism effectively ensures the insulation reliability of the material. The proposed interface engineering strategy provides a fundamental theoretical basis for designing high-performance epoxy insulating materials, with the multifunctional optimization approach being extendable to other nanocomposite dielectric systems.

## Figures and Tables

**Table 1:** The potential shift in Metal in the bulk region.

	Average Potential (eV)	Potential Shift (eV)
Cu-EP	−5.132	0
Cu-EP/SiO_2_	−5.566	−0.434
Fe-EP	−4.122	0
Fe-EP/SiO_2_	−4.381	−0.259

**Table 2:** The potential shift in Epoxy resin in the bulk region.

	Average Potential (eV)	Potential Shift (eV)
Cu-EP	2.025	0
Cu-EP/SiO_2_	1.025	−1.0
Fe-EP	1.482	0
Fe-EP/SiO_2_	0.393	−1.089

## Data Availability

All the data are available within the manuscript. Additional data will be provided upon request from the corresponding authors.

## Supplementary Materials

The following supporting information can be downloaded at: https://www.mdpi.com/article/10.3390/molecules30142960/s1. This supplementary material contains the Crystallographic Information Files (CIF) for all molecular structures as shown in Figure 2 and Figure 3.

## References

[1] Ohki Y. (2010). Development of Epoxy Resin Composites with High Thermal Conductivity. IEEE Electr. Insul. Mag..

[2] Capricho J.C., Fox B., Hameed N. (2019). Multifunctionality in Epoxy Resins. Polym. Rev..

[3] Dong M., Zhang H., Tzounis L., Santagiuliana G., Bilotti E., Papageorgiou D.G. (2021). Multifunctional Epoxy Nanocomposites Reinforced by Two-Dimensional Materials: A Review. Carbon.

[4] Wang W., Li R., Hu L., Jiang Q., Li S. (2024). Nonlinear Dielectric Response and Conductivity Characteristics of Epoxy Resin in High Voltage AC Electric Field. J. Phys. D Appl. Phys..

[5] Ren J.-W., Zeng R.-C., Yang J., Wang Z., Wang Z., Zhao L.-H., Wang G.-L., Jia S.-L. (2024). Significantly Enhancing the Through-Plane Thermal Conductivity of Epoxy Dielectrics by Constructing Aramid Nanofiber/Boron Nitride Three-Dimensional Interconnected Framework. J. Appl. Phys..

[6] Mi R., Xing Z., Hao J., Hu X., Min D., Li S., Wu Q. (2020). Effect of Morphology and Traps on DC Conductivity and Breakdown of Polyethylene Nanocomposites. IEEE Trans. Dielectr. Electr. Insul..

[7] Wang Y., Luo Y., Guan J., Ding R. (2019). Dielectric Properties of Epoxy Resin Impregnated Paper Insulation in Different Stages of Partial Discharge Development. Polym. Compos..

[8] Yu J., Ding S., Yu S., Lu Y.-C., Xu P., Chu B., Sun R., Xu J., Wong C.-P. (2020). Nanoparticles with Rationally Designed Isoelectronic Traps as Fillers Significantly Enhance Breakdown Strength and Electrostatic Energy Density of Polymer Composites. Compos. Sci. Technol..

[9] Yang K., Chen W., Zhao Y., Ding L., Du B., Zhang S., Yang W. (2022). Enhancing Dielectric Strength of Thermally Conductive Epoxy Composites by Preventing Interfacial Charge Accumulation Using Micron-Sized Diamond. Compos. Sci. Technol..

[10] Huang J., Lin F., Hin C. (2019). Toward a Unified Theory Correlating Electronic, Thermodynamic, and Mechanical Properties at Defective Al/SiO_2_ Nanodevice Interfaces: An Application to Dielectric Breakdown. ACS Appl. Nano Mater..

[11] Shi Y., Chen X., Meng F.-B., Hong Z., Awais M., Paramane A. (2022). Enhancement of Insulation Properties of Cross-Linked Polyethylene Utilizing Aromatic Voltage Stabilizers with Electron-Withdrawing and Electron-Donating Groups. ACS Appl. Polym. Mater..

[12] Shao S., Loi M.A. (2019). The Role of the Interfaces in Perovskite Solar Cells. Adv. Mater. Interfaces.

[13] Ren Y., Zhang Q., Wang Y., Li J., Yan W., Xu H., Cheng C., Wu K. (2024). A First-Principles Study of the Formation and Regulation of the Electric Double Layers at Cu (0 0 1)/Mineral Oil Interfaces. J. Phys. D Appl. Phys..

[14] Madonna V., Giangrande P., Galea M. (2021). Influence of Insulation Thermal Aging on the Temperature Assessment in Electrical Machines. IEEE Trans. Energy Convers..

[15] Wang T., Li D., Zhang G. (2021). Molecular Dynamics Simulations of Interface Properties and Key Physical Properties of Nanodielectrics Manufactured With Epoxy Resin Doped With Metal Nanoparticles. IEEE Access.

[16] Luo J., Yang X., Xue Y., Yang C., Yang Z., Tusiime R., Liu Y., Zhang H., Yu J. (2023). Simultaneous Optimization of the Thermal Conductivity and Mechanical Properties of Epoxy Resin Composites through PES and AgNP Functionalized BNs. Compos. Part B Eng..

[17] Sallal H.A., Abdul-Hamead A.A., Othman F.M. (2020). Effect of Nano Powder (Al_2_O_3_-CaO) Addition on the Mechanical Properties of the Polymer Blend Matrix Composite. Def. Technol..

[18] Patil S.P., Shendye P., Markert B. (2020). Molecular Dynamics Simulations of Silica Aerogel Nanocomposites Reinforced by Glass Fibers, Graphene Sheets and Carbon Nanotubes: A Comparison Study on Mechanical Properties. Compos. Part B Eng..

[19] Kai M.F., Zhang L.W., Liew K.M. (2020). Carbon Nanotube-Geopolymer Nanocomposites: A Molecular Dynamics Study of the Influence of Interfacial Chemical Bonding upon the Structural and Mechanical Properties. Carbon.

[20] Shiu S.-C., Tsai J.-L. (2014). Characterizing Thermal and Mechanical Properties of Graphene/Epoxy Nanocomposites. Compos. Part B Eng..

[21] Mai Y., Du B., Liu Q., Zhao Y., Yang W., Yan B. (2021). Influence of Micro@Nano-Al_2_O_3_ Structure on Mechanical Properties, Thermal Conductivity, and Electrical Properties of Epoxy Resin Composites. J. Electron. Mater..

[22] Kitichatpayak D., Makcharoen W., Vittayakorn N., Vittayakorn W. (2022). Influence of Various Nanofillers on Mechanical and Electrical Properties of Epoxy Resin Composites. Polym.-Plast. Technol. Mater..

[23] Zhang C., Liu Z., Wang X., Zhang Q., Xing W., Zhang T., Chi Q. (2024). Research on Molecular Dynamics and Electrical Properties of High Heat-Resistant Epoxy Resins. J. Chem. Phys..

[24] He J., Li L., Zhou J., Tian J., Chen Y., Zou H., Liang M. (2023). Ultra-High Modulus Epoxy Resin Reinforced by Intensive Hydrogen Bond Network: From Design, Synthesis, Mechanism to Applications. Compos. Sci. Technol..

[25] Liu X., Rao Z. (2020). A Molecular Dynamics Study on Heat Conduction of Crosslinked Epoxy Resin Based Thermal Interface Materials for Thermal Management. Comput. Mater. Sci..

[26] Xie Q., Fu K., Liang S., Liu B., Lu L., Yang X., Huang Z., Lü F. (2018). Micro-Structure and Thermomechanical Properties of Crosslinked Epoxy Composite Modified by Nano-SiO_2_: A Molecular Dynamics Simulation. Polymers.

[27] Akkermans R.L.C., Spenley N.A., Robertson S.H. (2020). Compass Iii: Automated Fitting Workflows and Extension to Ionic Liquids. Mol. Simul..

[28] Farzi N., Ebrahim M. (2024). Mechanical Properties and Glass Transition Temperature of Metal-Organic Framework-Filled Epoxy Resin: A Molecular Dynamics Study. Mater. Chem. Phys..

[29] Kohn W., Sham L.J. (1965). Self-Consistent Equations Including Exchange and Correlation Effects. Phys. Rev..

[30] Kresse G., Furthmüller J. (1996). Efficiency of Ab-Initio Total Energy Calculations for Metals and Semiconductors Using a Plane-Wave Basis Set. Comput. Mater. Sci..

[31] Kresse G., Hafner J. (1994). Ab Initio Molecular-Dynamics Simulation of the Liquid-Metal-Amorphous-Semiconductor Transition in Germanium. Phys. Rev. B.

[32] Kresse G., Hafner J. (1993). Ab Initio Molecular Dynamics for Liquid Metals. Phys. Rev. B.

[33] Xue Q., Lv C., Shan M., Zhang H., Ling C., Zhou X., Jiao Z. (2013). Glass Transition Temperature of Functionalized Graphene–Polymer Composites. Comput. Mater. Sci..

[34] Wang Y., Wang W., Zhang Z., Xu L., Li P. (2016). Study of the Glass Transition Temperature and the Mechanical Properties of PET/Modified Silica Nanocomposite by Molecular Dynamics Simulation. Eur. Polym. J..

[35] Feng R., Zhang X., Qing S., Zheng M., Wang H. (2023). Stability of Soluble Bulk Nanobubbles: Many-Body Dissipative Particle Dynamics Analysis. J. Mol. Liq..

[36] Li Y., Li H., Song C., Zhu Z., Ma X. (2024). Molecular Dynamics Simulations of the Micro Mechanism of Functionalized SiO_2_ Nanoparticles and Carbon Nanotubes Modified Epoxy Resin Adhesives. Polym. Compos..

[37] Ma L., Liu H., Wen X., Szymańska K., Mijowska E., Hao C., Tang T., Lei Q. (2022). Polyhydric SiO_2_ Coating Assistant to Graft Organophosphorus onto Glass Fabric for Simultaneously Improving Flame Retardancy and Mechanical Properties of Epoxy Resin Composites. Compos. Part B Eng..

[38] Fu K., Lü F., Xie Q., Ruan H., Yang X., Liang S. (2020). The Effects of Shape and Mass Fraction of Nano-SiO_2_ on Thermomechanical Properties of Nano-SiO_2_/DGEBA/MTHPA Composites: A Molecular Dynamics Simulation Study. AIP Adv..

[39] Liao B., Wu S., Yang L. (2017). Free Volume: An Indicator of the Glass-Forming Ability in Binary Alloys. AIP Adv..

[40] Wei Q., Zhang Y., Wang Y., Yang M. (2017). A Molecular Dynamic Simulation Method to Elucidate the Interaction Mechanism of Nano-SiO_2_ in Polymer Blends. J. Mater. Sci..

[41] Gaillac R., Pullumbi P., Coudert F.-X. (2016). ELATE: An Open-Source Online Application for Analysis and Visualization of Elastic Tensors. J. Phys. Condens. Matter.

[42] Zhou Y., Hu J., Dang B., He J. (2016). Titanium Oxide Nanoparticle Increases Shallow Traps to Suppress Space Charge Accumulation in Polypropylene Dielectrics. RSC Adv..

[43] Dang B., He J., Hu J., Zhou Y. (2016). Large Improvement in Trap Level and Space Charge Distribution of Polypropylene by Enhancing the Crystalline—Amorphous Interface Effect in Blends. Polym. Int..

[44] Wang Z.L. (2021). From Contact Electrification to Triboelectric Nanogenerators. Rep. Prog. Phys..

[45] Castle G.S.P., Schein L.B. (1995). General Model of Sphere-Sphere Insulator Contact Electrification. J. Electrost..

[46] Xu C., Zi Y., Wang A.C., Zou H., Dai Y., He X., Wang P., Wang Y.-C., Feng P., Li D. (2018). On the Electron-Transfer Mechanism in the Contact-Electrification Effect. Adv. Mater..

[47] Köhler L., Kresse G. (2004). Density Functional Study of CO on Rh(111). Phys. Rev. B—Condens. Matter Mater. Phys..

[48] Heyd J., Scuseria G.E., Ernzerhof M. (2003). Hybrid Functionals Based on a Screened Coulomb Potential. J. Chem. Phys..

[49] Straumann U., Schuller M., Franck C.M. (2012). Theoretical Investigation of HVDC Disc Spacer Charging in SF_6_ Gas Insulated Systems. IEEE Trans. Dielectr. Electr. Insul..

[50] Chi Q., Wang Y., Zhang C., Zhang Y., Zhang Y., Tang C., Zhang T. (2023). IEEE Transactions on Dielectrics and Electrical Insulation Information for Authors. IEEE Trans. Dielectr. Electr. Insul..

[51] Zhang B., Qi Z., Zhang G. (2017). Charge Accumulation Patterns on Spacer Surface in HVDC Gas-Insulated System: Dominant Uniform Charging and Random Charge Speckles. IEEE Trans. Dielectr. Electr. Insul..

[52] Li C., Hu J., Lin C., Zhang B., Zhang G., He J. (2017). Surface Charge Migration and Dc Surface Flashover of Surface-Modified Epoxy-Based Insulators. J. Phys. D Appl. Phys..

